# Recent advances in the structural and molecular biology of type IV secretion systems^☆^

**DOI:** 10.1016/j.sbi.2014.02.006

**Published:** 2014-08

**Authors:** Martina Trokter, Catarina Felisberto-Rodrigues, Peter J Christie, Gabriel Waksman

**Affiliations:** 1 Institute of Structural and Molecular Biology, University College London and Birkbeck, Malet Street, London WC1E 7HX, UK; 2 Department of Microbiology and Molecular Genetics, University of Texas Medical School, 6431 Fannin Street, Houston, TX 77030, USA

## Abstract

Bacteria use type IV secretion (T4S) systems to deliver DNA and protein substrates to a diverse range of prokaryotic and eukaryotic target cells. T4S systems have great impact on human health, as they are a major source of antibiotic resistance spread among bacteria and are central to infection processes of many pathogens. Therefore, deciphering the structure and underlying translocation mechanism of T4S systems is crucial to facilitate development of new drugs. The last five years have witnessed considerable progress in unraveling the structure of T4S system subassemblies, notably that of the T4S system core complex, a large 1 MegaDalton (MDa) structure embedded in the double membrane of Gram-negative bacteria and made of 3 of the 12 T4S system components. However, the recent determination of the structure of ∼3 MDa assembly of 8 of these components has revolutionized our views of T4S system architecture and opened up new avenues of research, which are discussed in this review.

## Highlights

• We describe the first structure of a type IV secretion (T4S) system.
• The previously reported core complex is mostly an outer membrane complex.
• We describe the newly discovered inner membrane complex and the stalk.
• We discuss proposed translocation mechanisms of T4S systems.
• We discuss the regulation of pilus biogenesis and substrate transfer by T4S systems.

**Current Opinion in Structural Biology** 2014, **27**:16–23

This review comes from a themed issue on **Membranes**

Edited by **Tamir Gonen** and **Gabriel Waksman**

For a complete overview see the Issue and the Editorial

Available online 5th April 2014

0959-440X/$ – see front matter, © 2014 The Authors. Published by Elsevier Ltd. All rights reserved.

**http://dx.doi.org/10.1016/j.sbi.2013.12.004**

## Introduction

Secretion in bacteria is the process by which macromolecules are translocated across the cell envelope. Gram-negative bacteria are confronted with an arduous task of substrate translocation across two cell membranes. To overcome this challenge, they have evolved a diversity of specialized secretion systems [1, 2]. Among six known types of secretion systems in Gram-negative bacteria, the type IV secretion (T4S) system is the most versatile.

T4S systems can be divided into three groups according to their function [3, 4]. The first group consists of conjugation systems that translocate single-stranded DNA substrates to recipient cells in a contact-dependent manner (promoting adaptation of bacteria to changes in their environment). The second group comprises effector translocation systems that deliver protein substrates directly to eukaryotic cells (such as virulence factors in the course of an infection). A third smaller group of T4S systems includes systems that mediate DNA uptake from the environment or release of DNA or protein substrates into the extracellular milieu independently of contact with another cell. The ability to transport nucleic acids in addition to proteins makes T4S systems unique among secretion systems.

In this review, we describe the recent breakthroughs in the structural biology of T4S systems and the proposed mechanisms of action of these complex machines.

## T4S system composition and general architecture

**Figure 1:**
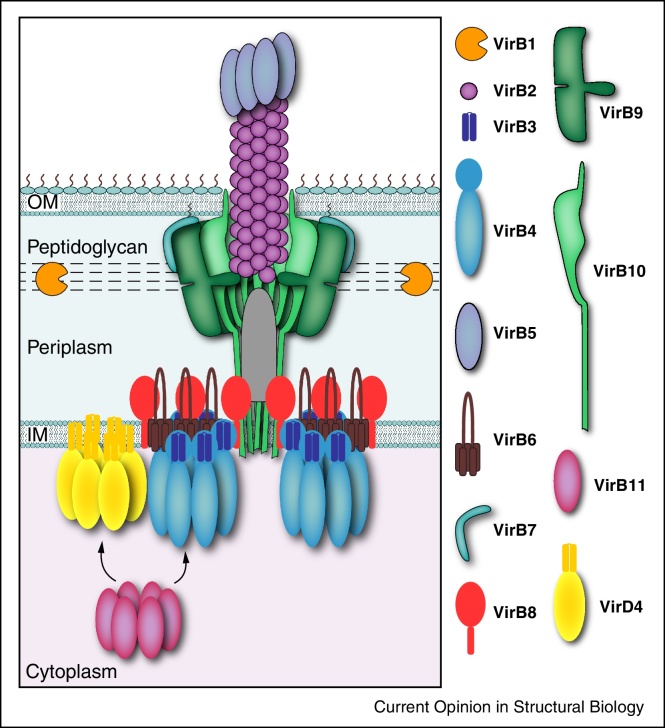
Schematic of the T4S system. Subunits at the right, identified with the *A. tumefaciens* VirB/VirD4 nomenclature, assemble as the T4S apparatus/pilus across the Gram-negative cell envelope. Hexameric ATPases establish contacts with the integral inner membrane (IM) subunits to form an inner membrane complex. VirB7, VirB9, and VirB10 form a core complex extending from the IM, periplasm, and outer membrane (OM). A domain of unspecified composition (grey bullet structure) and the pilus assemble within the central chamber of the core complex.

The most studied T4S systems are the VirB/D4 system from *Agrobacterium tumefaciens* and the closely related systems from *Escherichia coli* encoded by the conjugative plasmids F, R388, and pKM101 [3]. They consist of 12 proteins named VirB1–VirB11 and VirD4 (only *A. tumefaciens* nomenclature will be used herein). Many T4S systems found in Gram-negative bacteria are similar to the VirB/VirD4 system, although they may vary in subunit number and composition [4]. VirB3 and VirB6-B10 form the scaffold of a substrate translocation apparatus that spans both membranes (Figure 1), the architecture of which was not known until very recently [5^••^]. Apart from secreting substrates, the T4S apparatus is also used for biogenesis of an extracellular pilus that is composed of the pilin subunit VirB2 [6] and the pilus-tip adhesin VirB5 [7]. VirB1 is a periplasmic lytic transglycosylase that is thought to locally lyse the peptidoglycan layer [8], and is required for pilus biogenesis [9], but is not essential for the assembly of the translocation apparatus [10]. Three ATPases, VirB4, VirB11 and VirD4 power substrate translocation and pilus biogenesis.

**Figure 2:**
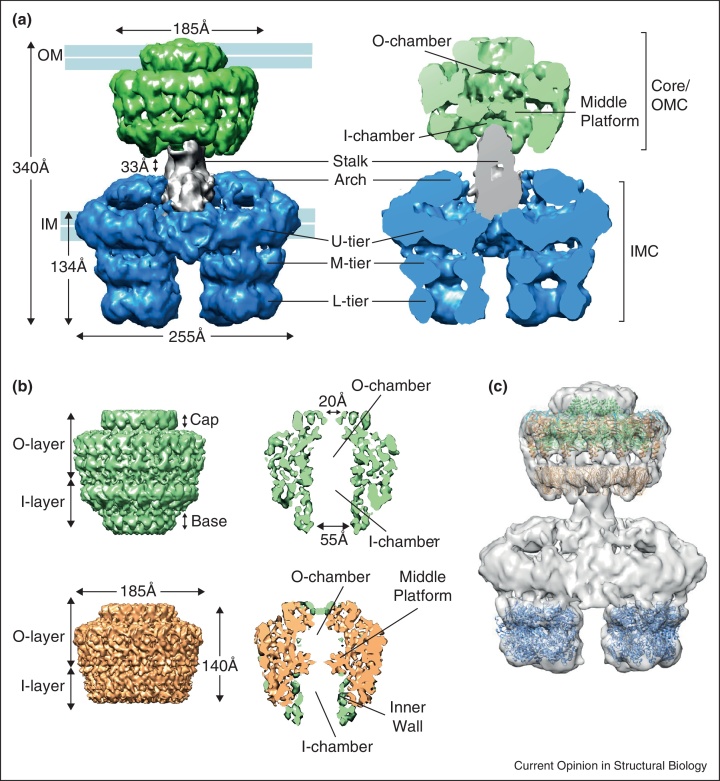
EM reconstructions showing the structure of the T4SS_3–10_ complex and the core complex. **(a)** Front view (left) and cut-away front view (right) of the T4SS_3–10_ complex (EMD-2567) comprising the core/outer membrane complex (core/OMC, green), the stalk (grey) and the inner membrane complex (IMC, blue). U-tier, M-tier and L-tier stand for upper, middle and lower tier, respectively. The inner (IM) and outer (OM) membranes are indicated. **(b)** pKM101 core complex (EMD-2232) (top) and truncated core complex lacking the N-terminal part of VirB10 (EMD-2233) (bottom): side view (left) and cut-away side view (right). The bottom right panel shows the superposition of the difference map (between the full-length and the truncated core complex cryo-EM maps) in green, and the cryo-EM structure of the truncated core complex in orange (as in bottom left). The VirB10 N-terminus forms the inner wall of the I-layer and the base. **(c)** T4SS_3–10_ complex with fitted crystal structures of the VirB4 C-terminal domain from *Thermoanaerobacter pseudethanolicus* (PDB: 4AG5) and the pKM101 outer membrane complex (PDB: 3JQO) and in silico model of the N-terminal domain of VirB9 from pKM101 (PDB: 3ZBJ).

Recently, the first structural characterization of a T4S apparatus composed of the VirB3-B10 subunits from the R388 plasmid (hereafter referred to as ‘the T4SS_3–10_ complex’; EMD-2567) has unraveled the architecture of T4S machines [5^••^]. The electron microscopy (EM) reconstruction obtained from the analysis of negatively stained single particles revealed a massive complex (∼3 MDa), 340 Å in length and 255 Å at its widest (Figure 2a). The structure showed the T4S system to be composed of: firstly, the clearly recognizable outer membrane (OM) complex, the core complex, of which a high-resolution structure (from pKM101 plasmid) has been previously resolved using cryo-EM and X-ray crystallography [11••, 12•, 13]; secondly, the previously uncharacterized inner membrane complex (IMC) of unprecedented architecture; and finally, a thin flexible region connecting the core complex and the IMC, the stalk.

## The core/outer membrane complex

Cryo-EM reconstruction of the core complex from pKM101 plasmid obtained by single particle analysis (EMD-5031, EMD-2232) revealed a ring-like structure of 185 Å in width and height (Figure 2b top) composed of the VirB7, VirB9 and VirB10 subunits, each present in 14 copies [11••, 13]. The complex formed two main layers, inner (I) and outer (O) layer. The O-layer, composed of the lipoprotein VirB7 and the C-terminal domains of VirB9 and VirB10, narrows at the top into a ‘cap’ structure with a small central opening of ∼20 Å and a constriction of ∼10 Å in diameter that inserts in the outer membrane (OM). The I-layer, composed of the N-terminal domains of VirB9 and VirB10, is widely open at the bottom with an aperture of 55 Å in diameter. The base of the I-layer was suggested to be formed by the VirB10 N-terminus, which was previously shown to insert into the inner membrane (IM) via its transmembrane (TM) helix [14]. As discussed in more detail below, it has now become evident that the VirB10 N-terminus folds here in a compact structure, forming the inner wall of the I-layer and a ring-like structure at its base [5^••^].

**Figure 3:**
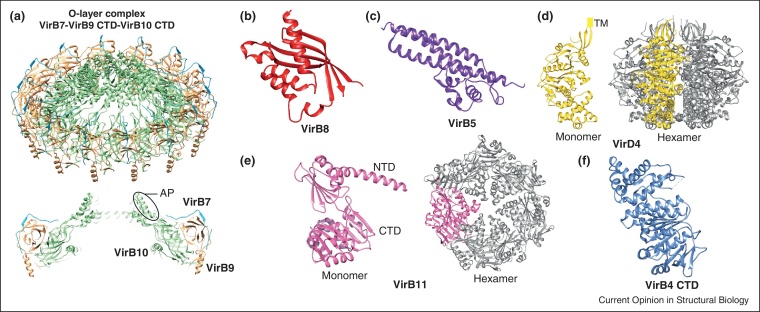
Crystal structures of the T4S system subunits and subassemblies. **(a)** pKM101 outer layer complex (PDB: 3JQO); **(b)** VirB8 periplasmic domain of *Brucella suis* (PDB: 2BHM); **(c)** VirB5 from pKM101 plasmid (1R8I); **(d)** cytoplasmic domain of VirD4 from R388 plasmid (PDB: 1GKI); **(e)** VirB11 homologue from *Helicobacter pylori* (PDB: 2PT7); **(f)** VirB4 C-terminal domain from *Thermoanaerobacter pseudethanolicus* (PDB: 4AG5).

The crystal structure of the O-layer (PDB: 3JQO; Figure 3a) revealed that VirB10 forms the entirety of the inner wall of the structure, whereas VirB7 and VirB9 wrap around it forming the outer wall of the O-layer [12^•^]. The cap structure is formed by 14 copies of a VirB10 domain comprising two α-helices separated by a loop, termed the antennae projection (AP), making VirB10 subunits unique bacterial proteins in their ability to span the entire cell envelope.

Recently, a cryo-EM structure of a truncated pKM101 core complex lacking the N-terminal part of VirB10 (EMD-2233; Figure 2b bottom) was solved at 8.5 Å resolution [13]. The I-layer of this complex contains only an outer wall, which is made of 14 VirB9 N-terminal domains. Using molecular modeling, the authors suggested that these domains are composed of β-sandwich folds (PDB: 3ZBJ). Interesting protrusions were observed that narrowed the passage between the chambers made by the O-layer and I-layer, forming a middle platform (Figure 2b bottom right). The middle platform, presumably made of the VirB9 central region, might be mediating substrate translocation through the core complex chamber.

The core complex retained its overall structure in the context of the recently determined T4SS_3–10_ structure (Figure 2a) [5^••^]. However, in the T4SS_3–10_ structure, the core complex is located on top of a large IMC, demonstrating that most of the core complex locates at and near the OM. Thus, except for the very N-termini of VirB10, which are known to insert in the IM and are part of the IMC, the core complex is primarily an ‘outer membrane complex’ (Figure 2a). As the IM is now thought to be located some distance away from the I-layer of the core complex, the VirB10 N-termini in the T4SS_3–10_ complex must adopt a much more extended conformation in comparison to their compact fold in the isolated core complex, in order to extend to the IM and form connections between the core/OM complex and the IMC (Figure 1). It is, however, important to note that the flexibility of this region did not allow tracking of 14 VirB10 N-termini extending from the core/OM complex. Thus, their precise location remains to be determined.

The middle platform was present in the core/OM complex of the T4SS_3–10_ complex as well (Figure 2a right). The composition of this middle platform remains unclear. It was hypothesized to be formed by some sequence of VirB9 between its two domains, but it could also include inter-domain sequences of VirB10, as VirB10 is known to form the inner wall of the entire core complex (Figure 2b) [13].

## The inner membrane complex

The recent reconstruction of the T4SS_3–10_ complex revealed for the first time the structure of the IMC (at 23 Å resolution) [5^••^], a massive assembly composed of VirB3, VirB4, VirB6, VirB8, and the VirB10 N-termini.

The IMC displays pseudo two-fold symmetry around the particle long axis (Figure 2a). The most prominent structures in the IMC are two barrels, one on each side of the complex, with a length of 134 Å and a minimum diameter of 105 Å. Gold-labeling experiments of VirB4 in combination with VirB4 stoichiometric determinations demonstrated that each barrel contains six VirB4 subunits, resembling the previously reported single particle EM reconstruction of VirB4 from the R388 plasmid [15]. Each barrel comprises three tiers: upper, middle and lower tier (Figure 2a). The middle and lower tiers are composed of VirB4 cytoplasmic C-terminal domains (Figure 2c), arranged as trimer of dimers, and form a ring with a central channel. The upper tier was suggested to be either partially or wholly inserted within the IM and is occluded (Figure 2a right). Directly above each IMC barrel lies a notable structure, the arch. On the basis of measured stoichiometry [5^••^], the upper tier and the arches of the IMC contain 12 copies of each of the VirB4 N-terminal domains, the VirB3 and the VirB8 proteins, 24 copies of VirB6, and 14 fragments of VirB10 N-termini containing the TM region.

VirB3 is a small inner-membrane protein that binds to VirB4, and, intriguingly, in some T4S systems, the two are fused into a single protein [3]. Interaction of VirB3 with VirB4 might assist membrane localization of VirB4. VirB8 subunits are bitopic proteins with a short cytoplasmic N-terminal domain, a TM region, and a large C-terminal periplasmic domain [16]. X-ray structures have been solved for the VirB8 periplasmic fragments of *Brucella suis* (PDB: 2BHM; Figure 3b) [17] and *A. tumefaciens* (PDB: 2CC3) [18]. VirB8 interacts with many other VirB proteins, including VirB4 and VirB10 [2], and is likely central for the assembly of the IMC. VirB6 proteins from *A. tumefaciens* and *Helicobacter pylori* are polytopic inner-membrane proteins with a periplasmic N-terminus, five TM segments, a large central periplasmic loop and a cytoplasmic C-terminus [19, 20]. Gold labeling of the VirB6 N-terminus demonstrated that it is located on the cytoplasmic side of the T4SS_3–10_ complex. This suggests that VirB6 in the R388 system might contain an additional TM region close to its N-terminus.

At present, it is not clear which subunit(s) form the arch, however, based on subunit topology, this will likely be the VirB6 periplasmic loop, and/or the VirB8 periplasmic domain.

## The stalk

Between the upper tier of the IMC and the core/OM complex lies a central, elongated structure, termed ‘the stalk’ (Figure 2a). This structure penetrates deep into the I-layer chamber of the core/OM complex and occludes the pore formed by the middle platform between the two layers of the core/OM complex (Figure 2a right). While the two other major parts of the T4SS_3–10_ complex have defined symmetry (14-fold for the core/OM complex, six-fold symmetry for each IMC barrel, and two-fold symmetry between the two IMC barrels), the stalk does not exhibit any evident symmetry. It is an elongated structure, but due to its flexibility, its definite structure remains unclear. Also still unclear is the composition of this region: it could be made of the VirB10 fragment that is immediately C-terminal to the TM sequence, 14 of which would have perhaps collapsed centrally to form the stalk. It could also contain VirB5, the stoichiometry of which was established to be 12 [5^••^]. In a complete T4S system, the stalk might form a VirB5/VirB2-containing pilus-nucleating center, with the VirB10 extended N-termini draping around it (see also below).

## Pilus

Pili are extracellular tubular polymers [21, 22] thought to promote an initial contact with recipient cells and subsequent formation of mating junctions [3, 23, 24]. However, the nature of these junctions is not known. Recent study has shown that conjugation can occur at considerable cell-to-cell distances and that conjugative pili serve as channels for ssDNA transfer during the process [25^••^]. Using the *Agrobacterium* T4S system, the isolation of ‘uncoupling’ mutations that block detectable pilus biogenesis while permitting efficient DNA transfer suggested that the formation of an intact pilus might not be required for substrate secretion [14, 26]. The expression of VirB2 and VirB5 is, however, essential [10]. These results suggested that the T4S system might exist in or transition between two states: a secretion-competent state and a pilus biogenesis-competent state, extending either a short or a long pilus, respectively [26].

As already mentioned, the pilus is composed of the major subunit, the VirB2 pilin [6] and the minor subunit, VirB5 [27]. VirB5 was shown to localize to the T-pilus tip in *A. tumefaciens* [7], suggesting an adhesive function for this protein. The X-ray structure of VirB5 from pKM101 plasmid (PDB: 1R8I; Figure 3c) has been solved [28]. VirB5 carries a signal peptide for export into the periplasm and it has been shown to interact with VirB8 and VirB10 [29]. VirB8 has been implicated in the formation of the VirB2–VirB5 nucleation complex [29], thereby promoting pilus biogenesis.

VirB2 proteins insert into the IM where they are being processed [30] and presumably retained. Upon an unknown signal, the pilin monomers are extracted from the IM, most likely by the VirB4 ATPase [31], and polymerized into the pilus. Pilus polymerization is likely nucleated within the T4S apparatus, but at which level is currently not known. It has been postulated that the VirB2 cylindrical conduit is enclosed within the core complex chamber [12^•^]. In view of the new structure, it could emanate from some structure in the IMC or from the stalk.

It is important to note that, in order to accommodate a much wider pilus [3], the core complex OM pore would have to considerably expand. Different diameters of the cap opening have been observed in cryo-EM and X-ray structures, supporting that such a possibility might exist [12•, 13].

## ATPases/energy centers

Three ATPases, VirB4, VirB11 and VirD4 are essential for substrate secretion, and two of them, VirB4 and VirB11, are required for pilus biogenesis [2]. All three ATPases function as hexamers. VirD4 is inserted in the IM via its N-terminal domain. A crystal structure of the cytoplasmic domain of VirD4 encoded by R388 plasmid (PDB: 1GKI; Figure 3d) was solved [32]. VirB11 is a soluble protein and crystal structures were solved for VirB11 homologues from *H. pylori* (PDB: 2PT7, 1NLZ; Figure 3e) [33, 34] and *B. suis* (PDB: 2GZA) [35]. Finally, a crystal structure of the VirB4 C-terminal domain from *Thermoanaerobacter pseudethanolicus* (PDB: 4AG5, 4AG6; Figure 3f) was solved and shown to be strikingly similar to the structure of VirD4 in spite of a very low sequence identity between the two proteins [36].

VirD4 is also termed type IV coupling protein (T4CP), as it is essential for the recruitment of substrates to the T4S apparatus. Essentially, nearly all T4S substrates carry a specific signal that is being recognized by T4CP [37, 38]. In the case of conjugation, the translocation signal is carried by the relaxase [38, 39], a protein that is covalently bound to ssDNA substrate and translocated with it [38, 40]. Because of its structural similarities with molecular motors, such as F1 ATPase or ring helicases, it has been postulated that T4CP might pump ssDNA substrate across the IM in an ATP-dependent manner [41]. Where the VirD4 protein would locate within the T4S system is unknown. Following the T4SS_3–10_ structure breakthrough, it is tempting to speculate that VirD4 could replace one of the two VirB4 barrels, locate on the side of a VirB4 barrel, or even form mixed hexamers with VirB4.

On the basis of its structural similarity to a number of AAA^+^ ATPases involved in trafficking [34], VirB11 might be involved in unfolding and translocation of protein substrates (such as the relaxase). Conformational changes caused by ATP-binding and hydrolysis have been documented [34]; however, whether these conformational changes are sufficient to force unfolding upon the substrate is unclear. VirB11 was also suggested to regulate substrate secretion and pilus biogenesis by interacting with VirD4 and VirB4, respectively, and modulating their activity [42]. Location of VirB11 within the T4S machinery is also unknown. All three ATPases were shown to interact with each other [42, 43], and have been proposed to stack on top of each other [44]. However, in the absence of a T4S system structure that includes VirD4 and VirB11, this remains speculative.

## Translocation pathway/mechanism

The best understood pathway of substrate translocation by T4S systems is the one utilized by *A. tumefaciens* VirB/VirD4 system. This system translocates oncogenic DNA (T-DNA; also covalently bound to a relaxase) and several effector proteins to plant cells [45]. A formaldehyde-crosslinking assay termed transfer DNA immunoprecipitation (TrIP) was used to identify close contacts between the T-DNA substrate and VirB/VirD4 subunits during translocation [46^••^]. Contacts were detected sequentially with VirD4, VirB11, VirB6, VirB8, and finally, VirB2 and VirB9 [19, 46••]. On the basis of these experiments, the following translocation route for nucleoprotein substrates was proposed: the DNA is first recruited by VirD4, and then transferred to VirB11. Next, the substrate is delivered to the IMC components VirB6 and VirB8, and then finally passed on to VirB2 and VirB9 for transfer through the OM. VirB4 and VirB10 appear to play important regulatory roles in this process. For example, VirB4 ATPase activity is required for transfer from VirB11 to VirB6/VirB8 [43]. VirB10 does not contact DNA, but regulates DNA hand-over from VirB6/VirB8 to VirB2/VirB9 [46^••^]. VirB10 acts as an energy sensor, sensitive to ATP-binding and hydrolysis by the cytoplasmic ATPases VirD4 and VirB11 [47]. By spanning both the inner and outer membranes, VirB10 is indeed in a unique position to regulate substrate transfer from the VirB6/VirB8 inner membrane components to the VirB2/VirB9 outer membrane ones.

**Figure 4:**
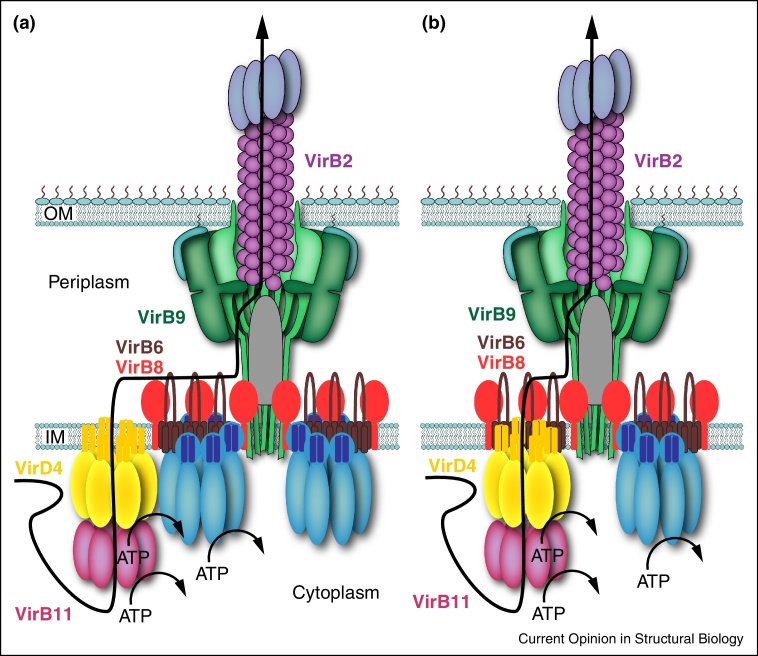
Schematic of the T4S system showing potential substrate translocation pathways: two-step mechanism **(a)** and one-step mechanism **(b)**. Only components of the *A. tumefaciens* VirB/D4 system that make contacts with T-DNA are indicated. See main text for details.

How can such a pathway be interpreted in view of the new T4SS_3–10_ structure? Unfortunately, this structure is missing two of the major components shown to interact with DNA: VirD4 and VirB11. In their absence, correlation between structure and function can only be speculative. In Low *et al.*, 2014, two VirD4-dependent pathways are proposed, both recapitulating already proposed mechanisms of substrate translocation by T4S systems (Figure 4). These two routes depend on where VirD4 might be located. VirD4 might be located on the side of the T4SS_3–10_ complex, and if correct, then a two-step mechanism of transport (as defined by [48]) must be invoked: the substrate would first be translocated through the IM (1st step) by VirD4 and then enter the core/OM complex through the periplasmic side of the complex (2nd step) thereby contacting the VirB6/VirB8 arches, then VirB9 (also contactable from the periplasmic side since it forms the outer wall of the core/OM complex), and finally entering the VirB2 pilus hypothesized here as forming a tube lining the entire VirB10-composed interior of the core/OM complex (Figure 4a). But VirD4 could also be located under the VirB4 barrels or substituting one of them. In that case, the substrate would go through the IMC of the T4SS_3–10_ structure, the arches, and then access the core/OM complex through contact with VirB9 and finally VirB2 as explained above (Figure 4b). How would VirB11 fit in is still unknown but location of VirD4 could be coupled to that of VirB4 in either scenario. Recently, VirB11 has been proposed to either interact with VirB4 to modulate the pilin-dislocase activity of VirB4 and thus participate together with VirB4 in pilus biogenesis, or interact with VirD4 to promote substrate transfer [42]. It is thus interesting to speculate that the T4SS_3–10_ structure might represent a T4S system in its pilus biogenesis mode, but substitution of the VirB4 barrels by VirD4 or formation of mixed VirB4/VirD4 barrels might switch the system to its substrate transfer mode.

## Conclusion

Although the T4SS_3–10_ structure represents a significant breakthrough in the field of secretion, there are still a considerable number of questions in need of answers. Where do VirD4 and VirB11 fit in the bigger context of the entire machinery? What does it take to induce a complex competent for pilus biogenesis, and one competent for substrate transfer and what is the structure of these two complexes? How is secretion being regulated by its various components? What is the substrate transfer pathway through the machinery? Those are essential questions that the field will aim to answer in the next few years. Given the alarming spread of antibiotics resistance, it is hoped that a thorough understanding of type IV secretion will emerge rapidly so that the process can be stopped at least in hospital setting where it is at its most devastating. To reach this goal, we have an arsenal of techniques available, including biochemical, biophysical and structural. It is by using a truly multidisciplinary approach with biochemistry at its heart that we will unlock the bottlenecks still to be faced in order to visualize either by EM or X-ray crystallography the structure of a fully assembled, fully functional T4S system.

## References and recommended reading

Papers of particular interest, published within the period of review, have been highlighted as:

• of special interest
•• of outstanding interest
